# The silver lining of a mind in the clouds: interesting musings are associated with positive mood while mind-wandering

**DOI:** 10.3389/fpsyg.2013.00583

**Published:** 2013-08-27

**Authors:** Michael S. Franklin, Michael D. Mrazek, Craig L. Anderson, Jonathan Smallwood, Alan Kingstone, Jonathan W. Schooler

**Affiliations:** ^1^Department of Psychological and Brain Sciences, University of California at Santa BarbaraSanta Barbara, CA, USA; ^2^Department of Psychology, University of California at BerkeleyBerkeley, CA, USA; ^3^Department of Social Neuroscience, Max Planck Institute for Human Cognitive Brain SciencesLeipzig, Germany; ^4^Department of Psychology, University of British ColumbiaVancouver, BC, Canada

**Keywords:** mind-wandering, mood, daydreaming, experience sampling, emotion

## Abstract

The negative effects of mind-wandering on performance and mood have been widely documented. In a recent well-cited study, Killingsworth and Gilbert (2010) conducted a large experience sampling study revealing that all off-task episodes, regardless of content, have equal to or lower happiness ratings, than on-task episodes. We present data from a similarly implemented experience sampling study with additional mind-wandering content categories. Our results largely conform to those of the Killingsworth and Gilbert (2010) study, with mind-wandering generally being associated with a more negative mood. However, subsequent analyses reveal situations in which a more positive mood is reported after being off-task. Specifically when off-task episodes are rated for interest, the high interest episodes are associated with an increase in positive mood compared to all on-task episodes. These findings both identify a situation in which mind-wandering may have positive effects on mood, and suggest the possible benefits of encouraging individuals to shift their off-task musings to the topics they find most engaging.

## INTRODUCTION

Mind-wandering, or having one’s attention diverted away from the current task, is such a common activity that estimates suggest nearly 30–50% of waking conscious experience is occupied by thoughts unrelated to a primary task (Klinger, 1999; Kane et al., 2007; Killingsworth and Gilbert, 2010; Franklin et al., 2011). The negative implications of mind-wandering have been widely documented across many different task contexts (see Mooneyham and Schooler, 2013 for a review) such as during reading (Schooler et al., 2004; Smallwood et al., 2008; Franklin et al., 2011), in tasks requiring sustained attention (e.g., the sustained attention to response task, SART; Smallwood et al., 2008), and learning and memory tasks (Smallwood et al., 2003; Mrazek et al., 2012a). Furthermore, mind-wandering is associated with negative mood, depression, attention deficit hyperactivity disorder (ADHD), and even shortened telomere length, a proxy of biological aging (e.g., Whalen et al., 2003; Smallwood et al., 2007c; Carriere et al., 2008; Smallwood and O’Connor, 2011; Epel et al., 2012).

Building on work showing that inducing negative mood increases mind-wandering (Smallwood et al., 2009) a large online experience sampling study by Killingsworth and Gilbert (2010) verified the relationship between negative well-being and mind-wandering. At random intervals throughout the day, participants were contacted via an iPhone (Apple Incorporated, Cupertino, CA, USA) application, and asked questions about their mind-wandering, current activities, thoughts, and emotions. Results revealed that overall, individuals’ reported reduced moods when they were mind-wandering relative to when they were on task. When they were thinking about neutral or negative topics their moods were particularly depressed, and regardless of current activity or the pleasantness of the mind-wandering episode, participants’ mood rating was always equivalent to or lower than on-task.

Although these findings confirm prior research showing that mind-wandering is typically associated with lower mood, there is also research that suggests that mind-wandering is not an entirely negative experience. For example, mind-wandering has been associated with enhanced creativity which could potentially boost mood. Baird et al. (2012) had participants perform an unusual uses test (i.e., “name all the possible uses for a nail”) with an intervening task that either promoted mind-wandering (an undemanding task) or did not (a demanding task). Subsequent performance on previously encountered problems was improved only for participants who engaged in a task that facilitated mind-wandering, presumably through a process of creative incubation. Given the association between positive affect and creativity (Amabile et al., 2005; Nadler et al., 2010), it seems plausible that creative musings may be an example of a class of mind-wandering that could be associated with positive mood.

We sought to examine whether there are particular types of mind-wandering associated with positive mood by using a more fine-grained assessment of the types of thoughts that people have during daily life. As part of a larger study to be reported elsewhere, participants from a community sample carried a personal digital assistant (PDA) with them for 1 week and were randomly probed throughout the day. In addition to being asked questions about mind-wandering and mood, participants were asked to rate their thoughts in terms of their usefulness, novelty, and interestingness. This study therefore allows us to examine (1) the general claim made by Killingsworth and Gilbert (2010) that all mind-wandering episodes have equal to or lower happiness ratings, than on-task episodes, and (2) whether mind-wandering episodes that rate high on these particular categories lead to enhanced positive mood relative to on-task episodes.

## MATERIALS AND METHODS

A total of 105 participants (71 female, mean age 23.1, SD = 7.4) were recruited by posted flyers on UBC campus and were paid $20 after the first session, $20 after the second session, and $30 for using the PDA. If participants responded to 75% or more of the experience sampling probes they went in a raffle for an extra $50.

Participants were provided with a PDA model Palm z22 and responded to the probes using the stylus. The Experience Sampling Program (ESP) was used to present the probes and collect data. Each time a participant was probed by the PDA they were first asked “Were you off-task?” If they responded yes, they were asked to rate their off-task thoughts on (1) how interesting were, (2) how useful they were, (3) how novel they were (that is, have you had identical thoughts previously) from 1 (not at all) to 5 (extremely). Then regardless of mind-wandering, all participants were asked to use the same five-point scale to answer (4) how positive is your mood at the moment and (5) how negative is your mood at the moment. Participants were also asked to rate awareness of mind-wandering, temporal focus, and how detrimental their mind-wandering was. These data were collected for a different project and were not analyzed for this study.

Participants were given a personal PDA to carry around which randomly probed participants and required them to respond to the questions described above approximately eight times per day during a 12-h interval pre-specified by the participant in which they would be available to respond to the probes.

## RESULTS

Overall, participants responded to 68.1% of the thought probes (a total of 3627 probes). Although this response rate is somewhat lower than Killingsworth and Gilbert’s (2010) sample (83%) it is similar to that reported by Kane et al. (2007; 72.5%) and McVay et al. (2009; 70.1%). Mind-wandering was reported for 26.2% of these responded to probes. While lower than proportion of time spent mind-wandering reported by Killingsworth and Gilbert (2010; 46.9%), this finding is in line with other experience sampling studies (Klinger, 1999; Kane et al., 2007). **Table 1** presents a correlation matrix of the content and mood variables.

**Table 1:** A correlation matrix of the content and mood variables.

	Detriment	Interest	Useful	Novel	Positive	Negative
Detriment
Interest	0.05
Useful	0.02	0.31^*^
Novel	0.06	0.19	0.14
Positive	-0.03	0.28^*^	0.14	0.08
Negative	0.17	-0.05	0.01	0.08	-0.44^*^

* *p* < 0.01.

**FIGURE 1:**
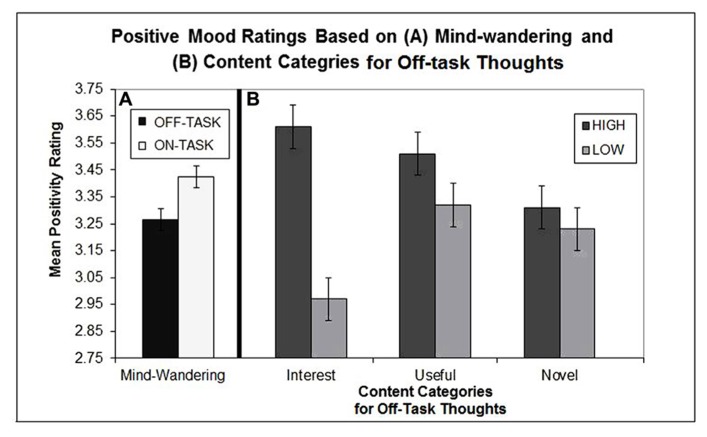
**(A)** Displays the mean positive mood ratings based on whether participants reported being on- vs. off-task for a given probe and **(B)** displays the mean positive mood ratings for off-task reports based on the three content categories participants were asked to use to rate their mind-wandering episode on. Error bars representing 95% confidence intervals are plotted for this figure using methods taken from Loftus and Masson (1994).

Subsequent analyses at the sample-level were done similarly to Killingsworth and Gilbert (2010; see supplemental methods) using a mixed effects model with a random intercept for subject (proc Mixed in SAS). This analysis increases power to detect effects in nested designs, making use of all the thought probe data while accounting for within subject correlations. First, we tested the general finding of Killingsworth and Gilbert (2010) that mind-wandering is associated with a less positive (“happy”) mental state. Consistent with the original findings, on-task reports had a higher positive mood rating (mean = 3.42, SD = 0.62) than off-task reports (mean = 3.27, SD = 0.63; *b* = 0.16, SE = 0.04; *F*(1,3502) = 18.84, *p* < 0.0001; see **Figure 1A**).

Next, we examined whether positive mood for a mind-wandering episode varied based on the three content categories measured for each episode (i.e., interest, usefulness, novelty). For this analysis, the middle rating scores were excluded (19.96%) and the data were re-coded as high (>3) or low (<3) in order to better segregate high from low ratings^1^. As seen in **Figure 1B**, positive mood ratings significantly differed based on this high/low grouping for interest [*b* = 0.71, SE = 0.09; *F*(1,586) = 68.82, *p* < 0.0001] and usefulness [*b* = 0.31, SE = 0.09; *F*(1,627) = 11.72, *p* < 0.001]. There was no effect of novelty (*p* = 0.93). These results suggest that mind-wandering content can influence mood and in particular mind-wandering episodes that are either high interest and/or high usefulness lead to increased positive mood relative to episodes that are low interest, low usefulness. Additional analyses revealed that high interest episodes were associated with a more positive mood than on-task episodes [*b* = 0.136, SE = 0.060; *F*(1,2850) = 5.16, *p* = 0.02] whereas highly useful episodes did not differ significantly from on-task episodes (*p* = 0.78). This result suggests that there are instances of mind-wandering associated with increased positive mood relative to being on-task

## DISCUSSION

Consistent with prior research, the present study indicates that mind-wandering is typically associated with lower mood. However, our results indicate that there are also instances when mind-wandering is associated with enhanced mood relative to being on-task. Specifically, high interest mind-wandering episodes were associated with an increase in positive mood relative to on-task reports.

The present findings have a number of implications from both basic and applied research perspectives. From a basic research perspective, these findings improve our understanding of the relationship between mind-wandering and mood. Whereas the clear negative implications of mind-wandering on mood have been reported previously (Watts et al., 1988; Smallwood et al., 2007a,b) and verified in the present study, the mood benefit of having interesting mind-wandering episodes points toward an important caveat to the reported link between mind-wandering and negative mood. Specifically, these results reveal that while overall mind-wandering is associated with reduced mood, there are certain topics of mind-wandering that may be associated with enhanced mood. Future research along these lines could help to shed light on the mechanism in which interesting mind-wandering and perhaps other ratings of mind-wandering (e.g., pleasantness) are associated with positive emotions. Future work will also be required to further clarify the causal relationship between mind-wandering mood, and interest.

In general, prior work has characterized interest as a positive state related to approach motivation (Fredrickson, 1998; Silvia, 2008). The emotion of interest has also been shown to replenish motivational resources which could enhance mood. Specifically, after completing a resource depleting task participants in conditions designed to increase feelings of interest persevered on an unrelated task for longer than participants in conditions that elicited just positive or negative emotion (Thoman et al., 2011). These findings illuminate a potential pathway by which interesting mind-wandering may benefit mood. Alternatively, it is possible that positive mood causes more interesting and useful thoughts. This perspective draws some support from research into the broaden-and-build theory of positive emotion. Specifically, positive mood is associated with broader thought–action repertoires and more diffuse attention (Fredrickson, 2001; Fredrickson and Branigan, 2005).

From an applied perspective, the present study provides an important counter-weight to the accumulating evidence that mind-wandering is problematic. As reviewed above, mind-wandering is widely linked with negative mood and with impaired performance across a range of tasks (Schooler et al., 2011). Within clinical settings, there are clear benefits of therapy aimed at reducing the frequency of negative ruminations in order to improve mood (Hofmann and Smits, 2008; Driessen and Hollon, 2010). Furthermore, the growing list of benefits associated with mindfulness (a state characterized by the absence of mind-wandering; Mrazek et al., 2012b) could be interpreted to suggest that mind-wandering is of no benefit. Without neglecting the seriousness of these negative associations, future research must also keep potential benefits of mind-wandering in view. As the present study indicates, there are circumstances in which diverting attention away from one’s current task is associated with a positive outcome at least with respect to its impact on mood. This view is consistent with an earlier framework developed by Singer and Antrobus (1963), who identified a “controlled thinking” type of daydreaming posited to be beneficial. Also, recent findings suggest that under some circumstances mind-wandering can promote future planning (Baird et al., 2011; Smallwood, 2013; Smallwood et al., 2013) and enhance creative incubation (Baird et al., 2012). The present study suggests that another important instance in which mind-wandering may be helpful: namely in the mood elevation that may arise from musing about particularly interesting topics.

Future research might profitably investigate the impact of asking individuals to identify topics that they find particularly interesting to think about, and then encouraging them to work at shifting the contents of their mind-wanderings toward those topics. The strategy of noticing negative mind-wandering episodes, or those related to ruminations regarding the past (Smallwood and O’Connor, 2011) and aiming to replace them with thoughts that are more interesting, positive and/or productive, is reminiscent of strategies that have been found to be effective in cognitive behavioral therapy (Beck, 1975; Clark and Beck, 1999). However, even with individuals who are not wrestling with depression, such a strategy might be helpful in both making mind-wandering episodes more productive, and reducing the negative mood that off-task thought otherwise typically induce. Moreover, if an experimental intervention that changed the content of mind-wandering episodes were found to improve the relationship between mind-wandering and mood, this would help clarify the causal direction of the relationship observed here. Clearly more research is needed to elucidate the potentially complex relationship between the contents of mind-wandering and mood. Nevertheless, the present results suggest that those of us who regularly find our minds in the clouds – musing about the topics that most engage us – can take solace in knowing that at least this form of mind-wandering is associated with elevated mood.

## Conflict of Interest Statement

The authors declare that the research was conducted in the absence of any commercial or financial relationships that could be construed as a potential conflict of interest.

## Footnotes

1 An analysis treating the variables as continuous yields identical results.
